# Genomic *in situ* hybridization in interspecific hybrids of scallops (Bivalvia, Pectinidae) and localization of the satellite DNA *Cf303*, and the vertebrate telomeric sequences (TTAGGG)n on chromosomes of scallop *Chlamys farreri* (Jones & Preston, 1904)

**DOI:** 10.3897/CompCytogen.v12i1.14995

**Published:** 2018-03-13

**Authors:** Liping Hu, Liming Jiang, Ke Bi, Huan Liao, Zujing Yang, Xiaoting Huang, Zhenmin Bao

**Affiliations:** 1 Ministry of Education Key Laboratory of Marine Genetics and Breeding, College of Marine Life Sciences, Ocean University of China, 5 Yushan Road, Qingdao 266003, China; 2 Yantai Fisheries Research Institute, Yantai 264003, China; 3 Museum of Vertebrate Zoology, University of California, Berkeley, California 94720, USA; 4 Laboratory for Marine Fisheries Science and Food Production Processes, Qingdao National Laboratory for Marine Science and Technology, Qingdao, China

**Keywords:** GISH banding, FISH, *Cf303*, telomere, scallop

## Abstract

Mitotic chromosome preparations of the interspecific hybrids *Chlamys farreri* (Jones & Preston, 1904) × *Patinopecten yessoensis* (Jay, 1857), *C. farreri* × *Argopecten irradians* (Lamarck, 1819) and *C. farreri* × *Mimachlamys nobilis* (Reeve, 1852) were used to compare two different scallop genomes in a single slide. Although genomic *in situ* hybridization (GISH) using genomic DNA from each scallop species as probe painted mitotic chromosomes of the interspecific hybrids, the painting results were not uniform; instead it showed species-specific distribution patterns of fluorescent signals among the chromosomes. The most prominent GISH-bands were mainly located at centromeric or telomeric regions of scallop chromosomes. In order to illustrate the sequence constitution of the GISH-bands, the satellite *Cf303* sequences of *C. farreri* and the vertebrate telomeric (TTAGGG)_n_ sequences were used to map mitotic chromosomes of *C. farreri* by fluorescence *in situ* hybridization (FISH). The results indicated that the GISH-banding pattern presented by the chromosomes of *C. farreri* is mainly due to the distribution of the satellite *Cf303* DNA, therefore suggesting that the GISH-banding patterns found in the other three scallops could also be the result of the chromosomal distribution of other species-specific satellite DNAs.

## Introduction

Chromosomal bandings were used to identify chromosomes and provided insight into the substructure and organization of whole chromosomes (Rønne 1990). Some chromosome banding techniques have been applied to bivalves, e.g. C-banding was used for investigating the distribution and composition of heterochromatin (Pasantes et al. 1996, Insua et al. 1998, Pauls and Affonso 2000, Huang et al. 2007b, García-Souto et al. 2016), silver staining was used for localizing the nucleolus organizer regions (Ag-NORs) (Martínez-Expósito et al. 1997, Pauls and Affonso 2000, Boroń et al. 2004), fluorescence counterstaining with chromomycin A_3_ or DAPI/PI used to identify the GC-rich or AT-rich heterochromatic regions (Martínez et al. 2002, Huang et al. 2007b, Zhang et al. 2007c, Pérez-García et al. 2010a, García-Souto et al. 2015, 2016), and fluorescent *in situ* hybridization (FISH) were used to localize 28S rDNA, 5S rDNA, and histone H3 gene to study karyotypic evolution on a variety of bivalves (Insua et al. 1998, 2006, Wang and Guo 2004, López-Piñón et al. 2005, Huang et al. 2006, 2007a, Odierna et al. 2006, Zhang et al. 2007b, Hu et al. 2011, Li et al. 2016, Yang et al. 2016, García-Souto et al. 2015, 2016). In addition, FISH was proved to be a valuable tool for mapping vertebrate telomere sequence (TTAGGG)_n_ on chromosomes of some bivalves (Wang and Guo 2001, Huang et al. 2007a, 2007b, García-Souto et al. 2016).

Genomic *in situ* hybridization (GISH) has been successfully used for discrimination of genome constitutions in hybrids and allopolyploids (Schwarzacher et al. 1989, 1992; Heslop-Harrison and Schwarzacher 1996; Hae-Woonet et al. 2008; Huang et al. 2011; Hu et al. 2013) and estimating phylogenetic relationships (Markova et al. 2007, Zhou et al. 2008). GISH-banding was occasionally observed on chromosomes with conventional GISH, which revealed chromosomal distribution of the randomly repeated sequences (Zhou et al. 2010). Patterns of GISH-banding coincident with those of Giemsa C-banding were first reported in the genus *Alstroemeria* (Linnaeus, 1762) using standard GISH with blocking DNA (Kuipers et al. 1997). Belyayev et al. (2001) constructed a GISH-banding karyotype of *Aegilops speltoides* (Tausch, 1837) and investigated the evolutionary dynamics of repetitive sequences in *Aegilops* (Linnaeus, 1753). Zhou et al. (2008) developed a GISH-banding protocol, built a universal reference karyotype of the *Secale cereale* (Linnaeus, 1753) chromosome 1R to 7R, and discriminate the repetitive sequence polymorphism in species or subspecies of *Secale*. In Pectinidae, GISH was used to discriminate parental genomes in hybrids and some GISH-bandings were observed which implied the different distribution of repetitive sequences (Huang et al. 2011, Hu et al. 2013, Huang et al. 2015). Recently, the genome of Yesso scallop, *Patinopecten yessoensis* (Jay, 1857) was sequenced and assembled, providing a thorough overview of the repetitive sequences constitution in Pectinidae (Wang et al. 2017). In addition, by paired-end sequencing of 2016 fosmid clones, a total of 2500 tandem repeats of *Chlamys farreri* (Jones & Preston, 1904), including 313 satellites, 1816 minisatellites and 371 microsatellites, were described (Zhang et al. 2008). However, the distributions of repetitive DNA sequences in chromosomes of different species of the family Pectinidae are still uncertain.

Repetitive DNA refers to DNA sequences that occur in multiple copies and makes up the major proportion of the nuclear DNA in most eukaryotic genomes. Changes in repetitive DNA likely contribute to the karyotypical features and variations, as well as genome sizes (Flavell 1986, San Miguel and Bennetzen 1998). Repetitive DNAs usually evolve faster than coding regions, and their sequence divergence may reflect evolutionary distances between species (Belyayev and Raskina 1998). Satellite DNAs, as the tandem arrays of repeated units, are paramount among repetitive sequences and can be located in centromeric, intercalary and/or subtelomeric chromosomal regions (Plohl et al. 2008, Plohl 2010, García-Souto et al. 2017), which are chiefly heterochromatic regions of chromosomes (Brutlag 1980).

In the present study, GISH was carried out on chromosomal slides of interspecific hybrids *C. farreri* × *P. yessoensis*, *C. farreri* × *Argopecten irradians* (Lamarck, 1819) and *C. farreri* × *Mimachlamys nobilis* (Reeve, 1852). Chromosomal distributions of the randomly repeated DNA sequences were revealed by GISH-banding in the four scallop species (*C. farreri*, *P. yessoensis*, *A. irradians* and *M. nobilis*). In order to verify the sequences constitution of GISH-banding, FISH with the satellite DNA *Cf303* and vertebrate telomere sequence (TTAGGG)_n_ probes were performed to compare the GISH-banding of *C. farreri*. Our results provided the first application of GISH-banding in Pectinidae, and first physical mapping of the satellite DNA *Cf303* and vertebrate telomere sequence (TTAGGG)_n_ in *C. farreri*, aiding to understanding chromosome distribution and composition of the repetitive DNA sequences in the studied scallops.

## Material and methods

### Specimens and chromosome preparations

The sexually mature scallops, *C. farreri*, *P. yessoensis*, *A. irradians* and *M. nobilis*, were obtained from hatcheries in Shandong Province, China. The interspecific hybrids *C. farreri* × *P. yessoensis*, *C. farreri* × *A. irradians* and *C. farreri* × *M. nobilis* were carried out in the laboratory. Eggs and sperm were collected from sexually mature scallops. Eggs were fertilized by adding sperm suspension. After fertilization, excessive sperm was removed by rinsing with seawater on a 20 µm screen (Wang and Wang 2008). The progeny individuals were sampled at the trochophore stage. Chromosome preparations were performed following the method of Zhang et al. (2007a). Briefly, following a treatment with colchicine (0.01 %, 1.5 h) and KCl (0.075 M, 20 min), trochophores were fixed three times (15 min each) in fresh ethanol/ glacial acetic acid solution (3:1 v/v). The fixed larvae were dissociated in 50 % acetic acid to obtain a cell suspension and that was then dropped onto hot-wet glass slides. The chromosome preparations were air-dried and stored at -20 ºC until use.

### Probe preparation

Telomeric (TTAGGG)_7_ probes were synthesized and 5’-end labelled with biotin-16-dUTP (Invitrogen). Plasmids were isolated from a fosmid clone containing the satellite DNA *Cf303* by standard laboratory methods (Sambrook et al. 1989). Genomic DNA was extracted from adductor muscle tissue using a standard phenolchloroform procedure (Sambrook et al. 1989). Then they were both labeled with biotin-16-dUTP by nick translation (Nick translation kit, Roche) following manufacturer’s instructions. The lengths of the DNA fragments were estimated by 2 % agarose gels and 100–600 bp were suitable as the probes for next GISH analysis.

### GISH and FISH

GISH and FISH were performed according to the methods of Huang et al. (2011) and Zhang et al. (2007a). Detection of biotin-labeled probes was carried out with fluorescein avidin DCS (Vector). Chromosomes were then counterstained with PI (Vector). Slides were examined with a Nikon Eclipse-600 epifluorescence microscope equipped with a CCD camera, and the signals were collected using appropriate filter sets for FITC and PI. The digital images were analyzed with a Lucia-FISH Image System Software. At least 10 complete metaphase chromosome spreads were examined for each sample.

## Results

**Figure 1.**
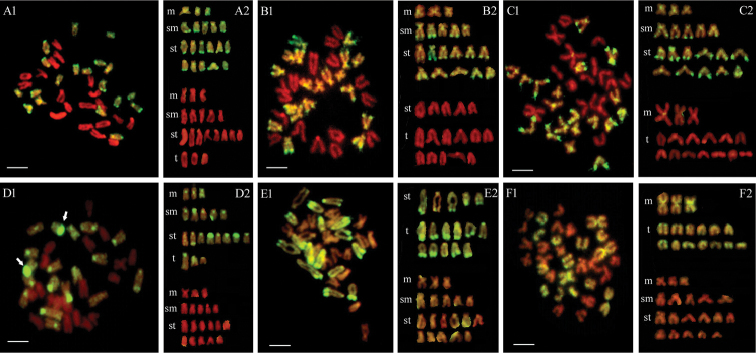
Metaphase chromosomes and karyotypes of scallop hybrids. **A1, A2, D1, D2** *C. farreri* × *P. yessoensis* **B1, B2, E1, E2** *C. farreri* × *A. irradians* **C1, C2, F1, F2** *C. farreri* × *M. nobilis* **A1, A2, B1, B2, C1, C2** the chromosomes originating from *C. farreri* were painted in yellow-green using the labeled genomic DNA probes from *C. farreri* **D1, D2** - the chromosomes originating from *P. yessoensis* were painted in yellow-green using the labeled genomic DNA probes from *P. yessoensis*. Nucleolus organizer regions (NORs) in *P. yessoensis* were marked with arrows in (**D1**). **E1, E2** the chromosomes originating from *A. irradians* were painted in yellow-green using the labeled genomic DNA probes from *A. irradians*. **F1, F2** the chromosomes originating from *M. nobilis* were painted in yellow-green using the labeled genomic DNA probes from *M. nobilis*. Scale bars: 5µm.

Using labeled total genomic DNA from *C. farreri* as probe, the hybridization signal (yellow-green) covered only chromosomes of *C. farreri*, but not *P. yessoensis*, *A. irradians* and *M. nobilis*, on the mitotic chromosomes of *C. farreri* hybrids with these scallop species (Fig. 1A1, B1, C1). Moreover, the signals on chromosomes of *C. farreri* were not uniform. The brighter fluorescent signals (GISH-bandings) clustered in the centromeric and/or peri-centromeric regions of 11–12 subtelocentric chromosomes, and the telomeric and/or peri-telomeric regions of the long arms of 13–14 submetacentric or subtelocentric chromosomes of *C. farreri*. In addition, the signal in telomeric regions was apparently stronger than that in the centromeric regions. Similarly, the hybridization signals only covered chromosomes of one parent whose genomic DNA was labeled as probes: the metaphase of *C. farreri* × *P. yessoensis* - by genomic DNA from *P. yessoensis* (Fig. 1D1), the metaphase of *C. farreri* × *A. irradians* - by genomic DNA from *A. irradians* (Fig. 1E1) and the metaphase of *C. farreri* × *M. nobilis* - by genomic DNA from *M. nobilis* (Fig. 1F1). Interestingly, the GISH-bandings of different scallops were significantly different. The GISH-bandings of *P. yessoensis* (Fig. 1D1, D2) were mainly distributed on centromeric regions of almost all chromosomes, the nucleolus organizer regions (NORs) where the signals were particularly strong in the peri-telomeric regions of the short arms of two subtelocentric chromosomes (arrows in Fig. 1D1), and peri-telomeric region of two subtelocentric chromosomes. The chromosomes of *A. irradians* were clearly labeled by the brighter signals in telomeric and/or peri-telomeric regions of the long arms of all chromosomes, in the short arms of two subtelocentric or telocentric chromosomes, and in interstitial regions of one chromosome (Fig. 1E1, E2). On the chromosomes of *M. nobilis*, the signals were dispersed along all them and some specific signals were located on centromeric regions (Fig. 1F1, F2).

**Figure 2.**
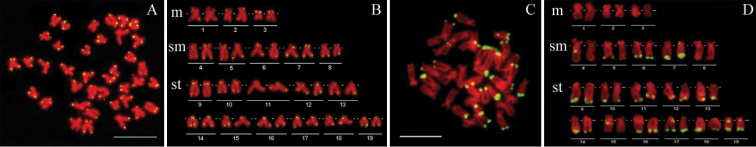
Metaphase chromosomes and karyotype of *C. farreri*. FISH mapping with vertebrate telomeric sequence (TTAGGG)_7_ (**A, B**) and satellite DNA *Cf303* (**C, D**). Scale bars: 5µm.

Considering that the GISH-bandings were mainly discovered in the telomeric and/or peri-telomeric regions of some chromosomes in *C. farreri*, the vertebrate telomeric sequence (TTAGGG)_n_, as well as satellite DNA *Cf303* were used as probes to compare the signal distribution. Telomeric repeats were hybridized to the ends of all *C. farreri* chromosomes, the signal intensity was weak and varied among different chromosomes, and no interstitial hybridization signal was observed (Fig. 2A, B). Satellite DNA *Cf303*, detected by FISH, were located on the centromeric region of a pair of subtelocentric chromosomes, and the telomeric regions of the long arms of 13–14 pairs of submetacentric or subtelocentric chromosomes in *C. farreri*, as shown in Fig. 2C, D.

## Discussion

Constitutive heterochromatin has been defined as a structurally distinct kind of chromatin comprising noncoding, largely repetitive DNA, which is permanently not transcribed (John 1988). The constitutive heterochromatic regions were usually detectable by C-banding (Sumner 1990, Sharma and Raina 2005). All C-bands correspond to heterochromatin but some heterochromatin was not stained by C-banding methods (Sumner 1990). In bivalves, the C-banding techniques were carried out in many species, such as mussels (Vitturi et al. 2000, Boroń et al. 2004), scallops (Insua et al. 1998, Pauls and Affonso 2000, Huang et al. 2007b) and oysters (Leitão et al. 2001, Pereira et al. 2011). Moreover, the C-banding patterns obtained in some species were consist with the chromomycin A3 positive bands or 4’6’-diamidino-2-phenylindole (DAPI) / propidium iodide (PI) banding patterns, revealing the GC-rich or AT-rich heterochromatic regions on chromosomes of bivalves (Boroń et al. 2004, Huang et al. 2007b, Zhang et al. 2007c, Petrović et al. 2009). However, C-bandings were not stable and still couldn’t be obtained successfully in many bivalve species (Xu et al. 2011).

Using GISH, Kuipers et al. (1997) found that GISH-banding patterns coincided with Giemsa C-banding patterns in the genus *Alstroemeria*. The consistent results between GISH-banding patterns and Giemsa C-banding patterns had been attributed to specific repetitive sequences, such as a tandem repeat from *Allium fistulosum* (Linnaeus, 1753), which was found to occur in major heterochromatic blocks (Irifune et al. 1995). FISH results of several highly repetitive sequences showed a significantly correspondence with the C-banding pattern in comparable studies of *S. montanum* (Gussone, 1825) (Cuadrado and Jouve 1995). The researches mentioned above were mainly focused on plant. However, the GISH-bandings in animals were rarely reported. In the present study, we observed significant GISH-bandings on the chromosomes of *C. farreri*, *P. yessoensis* and *A. irradians* after GISH. The results suggested the distribution of repetitive DNA (heterochromatin regions) were multifarious in scallops. In addition, the GISH-banding results of *P. yessoensis* and *A. irradians* corresponded roughly to their C-band-like patterns revealed by PI staining (Zhang et al. 2007c). Because there wasn’t reported about the herterochromatic region in chromosomes of *C. farreri*, we speculated that the GISH-bandings may reveal heterochromatic regions in *C. farreri*.

Highly repeated DNA exists within each eukaryotic genome. Satellite DNAs, as the tandem arrays of repeated units, are chiefly localized at heterochromatic regions of chromosomes (Brutlag 1980). The vertebrate telomeric repeat has been located at chromosome ends in some bivalves (Wang and Guo 2004, Huang et al. 2007a, García-Souto et al. 2016). To verify the constitution of GISH-banding, vertebrate telomeric sequences (TTAGGG)_n_ and satellite DNA *Cf303* were selected as probes to locate on chromosomes of *C. farreri* by FISH.

Vertebrate-type telomeric sequences (TTAGGG)_n_ located at terminal regions of each chromosome of *C. farreri* in our study. The signal intensities were weak and varied among different chromosomes; no interstitial hybridization signal was observed. This is the first report about the chromosomal distribution of telomeric sequences in *C. farreri*. The locations of these sequences were coincident with the results reported in *P. yessoensis* and *A. irradians* (Huang et al. 2007a, 2007b). In other bivalves, mostly terminal signals for these sequences were located in mussels (Mytilidae) (Martínez-Expósito et al. 1997, Plohl et al. 2002, Pérez-García et al. 2010a, 2010b, 2011), oysters (Ostreidae) (Guo and Allen 1997, Wang et al. 2001), wedge-shells (Donax) (Plohl et al. 2002, Petrović et al. 2009) and trough shells (Mactridae) (García-Souto et al. 2016, 2017). Indicia of intercalary signals were only reported in species of genus Mytilus (Martínez-Expósito et al. 1997, Plohl et al. 2002) and genus Brachidontes (Pérez-García et al. 2010b), probably as a result of the interspersion of telomeric sequences and the subterminal major rDNA. The telomeric signal intensity varied among different chromosomes in *C. farreri*, which suggested the length of telomeric repeat sequences were different among different chromosomes.

Satellite DNAs are highly repetitive DNA sequences that can be located in pericentromeric (Plohl et al. 1998), telomeric regions (Petrović et al. 2009) or intercalary regions (García-Souto et al. 2017). In bivalves, a highly repetitive satellite sequence *Cg*170 was located on the centromeric regions of 7 chromosomes in the *Crassostrea gigas* (Thunberg, 1793) (Wang et al. 2001). Our FISH results showed that the satellite DNA *Cf303* was located in centromeric region of one or two subtelocentric chromosome and the telomeric regions of the long arms of most submetacentric or subtelocentric chromosomes in *C. farreri*. Satellite DNAs could act as informative cytogenetic markers for the identification of chromosomal abnormalities, pairs of homologous chromosomes and specific regions of chromosomes, such as the α satellites of human (Gusella et al. 1982, Looijenga et al. 1990), CL1 and CL25 satellite repeats of *Raphanus sativus* (Linnaeus, 1753) (He et al. 2015), and the *Cg*170 satellites of *C. gigas* (Clabby et al. 1996). In this study, the satellite DNA *Cf303* could be developed as specific probe for identification of chromosomes in *C. farreri*.

In contrast to the location of telomere sequence in scallops, the satellite DNA *Cf303* existed only on the chromosomes of *C. farreri*, but not on the chromosomes of closely related species *P. yessoensis*, *A. irradians* and *M. nobilis*, which suggested that the satellite DNA *Cf303* was species-specific. After comparing the signal distribution and intensity of GISH-banding, vertebrate telomeric sequences and satellite DNA *Cf303*, we found the GISH-banding pattern in *C. farreri* was not consistent with the result of telomeric repeats. Interestingly, the GISH-bands overlapped the FISH signals obtained with satellite DNA *Cf303*. Generally, satellite DNAs are chiefly localized at heterochromatic regions of chromosomes (Brutlag 1980). Our results suggested that satellite DNA *Cf303* may represent the dominating component of heterochromatic regions in the chromosomes of *C. farreri*, as shown by GISH-banding.

Zhou et al. (2008) believed GISH-banding has displayed rapidly evolving repetitive sequences in the study of repetitive sequences polymorphism in *S. cereale*. In view of this, we speculated that the GISH-banding patterns in *C. farreri* were ascribed to rapidly evolving repetitive sequences, especially satellite DNA *Cf303*. In addition, the GISH-banding patterns of *P. yessoensis*, *A. irradians* and *M. nobilis* were completely different with that of *C. farreri*, which indicated that the repetitive sequences in the GISH-banding regions were distinctly species-specific in different scallop species. These species-specific satellite DNA and GISH-banding patterns would represent a useful tool in the scallop taxonomy for closely related species studies. Results of this study would further contribute to a better understanding the characteristics of genomic structure and to assess the evolutionary relationships within Pectinidae.
